# Lung adenocarcinoma with EGFR gene mutation metastatic to the uterine cervix: A case report

**DOI:** 10.1097/MD.0000000000022636

**Published:** 2020-10-16

**Authors:** Yong Wang, Lin Chen, Zhi Wang, Shubin Liu

**Affiliations:** a Department of Medical Oncology, The First Affiliated Hospital of Nanchang University, 17 Yongwai Zheng Road, Nanchang; b Department of Medical Oncology, The Affiliated Ganzhou Hospital of Nanchang University (Ganzhou People's Hospital), 16 Meiguan Road, Ganzhou; c Department of Internal Neurology; d Department of Pathology, The Affiliated Ganzhou Hospital of Nanchang University (Ganzhou People's Hospital), 16 Meiguan Road, Ganzhou, China.

**Keywords:** adenocarcinoma, EGFR mutation, lung cancer, metastasis, next-generation sequencing, uterine cervix

## Abstract

**Introduction:**

The cervix is a rare site of metastasis from advanced lung adenocarcinoma. Driven gene detection is particularly important for the treatment of advanced lung adenocarcinoma.

**Patient concerns:**

A 49-year-old Chinese female was sent to our hospital because of lumbago and sacroiliac joint pain; she was unable to walk and had vaginal bleeding. The following examinations were performed: imaging, colposcopy, bronchoscopy, immunohistochemistry and next-generation sequencing.

**Diagnosis:**

According to the clinical manifestations and the examination results, the diagnosis was lung adenocarcinoma with cervical, brain, adrenal gland and bone metastases. More importantly, EGFR gene mutations (del19) were detected in both the primary lung lesion and uterine cervical biopsy specimen.

**Interventions:**

Osimertinib was chosen as the first-line treatment.

**Outcomes:**

Lumbago and sacroiliac joint pain were significantly relieved. The levels of tumor markers decreased. Primary injuries and metastatic sites were significantly reduced.

**Conclusion:**

Physicians should be alert to the signals of vaginal bleeding and consider that primary lung adenocarcinoma may metastasize to the uterine cervix.

## 1 Introduction

Cervical cancer is generally metastatic to the lung via the blood circulation, but metastasis to the uterine cervix from primary lung carcinoma is rarely observed.^[1]^ Non-small-cell lung cancer (NSCLC) is generally metastatic to the regional lymph nodes, brain, bone, liver, and adrenal gland. The detection of mutations in and site distribution of NSCLC-related genes is particularly important for advanced lung adenocarcinoma. EGFR is the most commonly mutated gene in advanced NSCLC. Here, we first report a case of primary lung adenocarcinoma with EGFR gene mutation (del19) with metastasis to the uterine cervix. The patient provided informed consent for the publication of this case.

## 2 Case report

A 49-year-old nonsmoking Chinese female was sent to our hospital in June 2019 because of lumbago and sacroiliac joint pain; she was unable to walk, which had lasted for 3 weeks. She complained of vaginal bleeding with fatigue that had continued for approximately 1 month after experiencing amenorrhea for half a year. She did not have any other respiratory or nervous system symptoms, such as cough, hemoptysis, chest pain, dyspnea, or headache.

**Figure 1:**
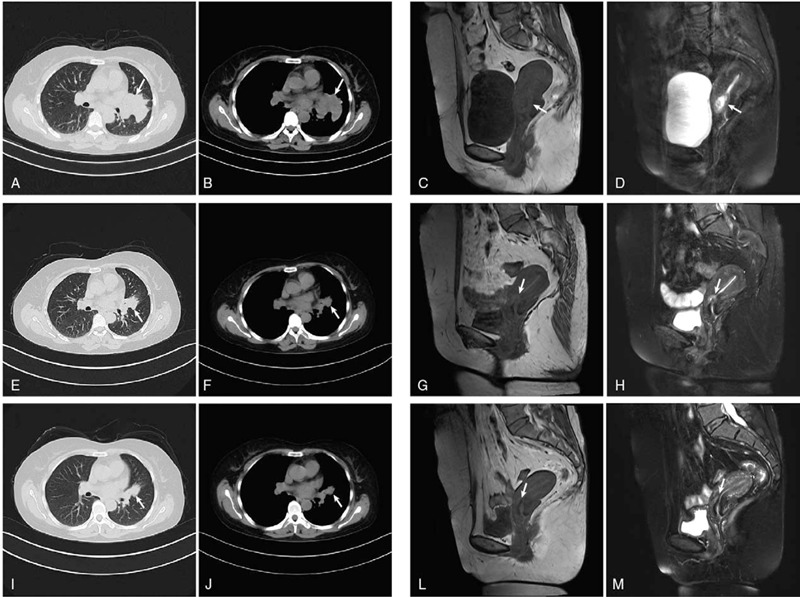
Axial CT demonstrates an irregular mass within the upper lobe of the left lung (A, B) one month (E, F) and 2 months (I, J) after treatment with osimertinib (white arrows). Sagittal MRI reveals a cervical mass lesion (C, D) one month (G, H) and 2 months (L, M) after treatment with osimertinib (white arrows).

**Figure 2:**
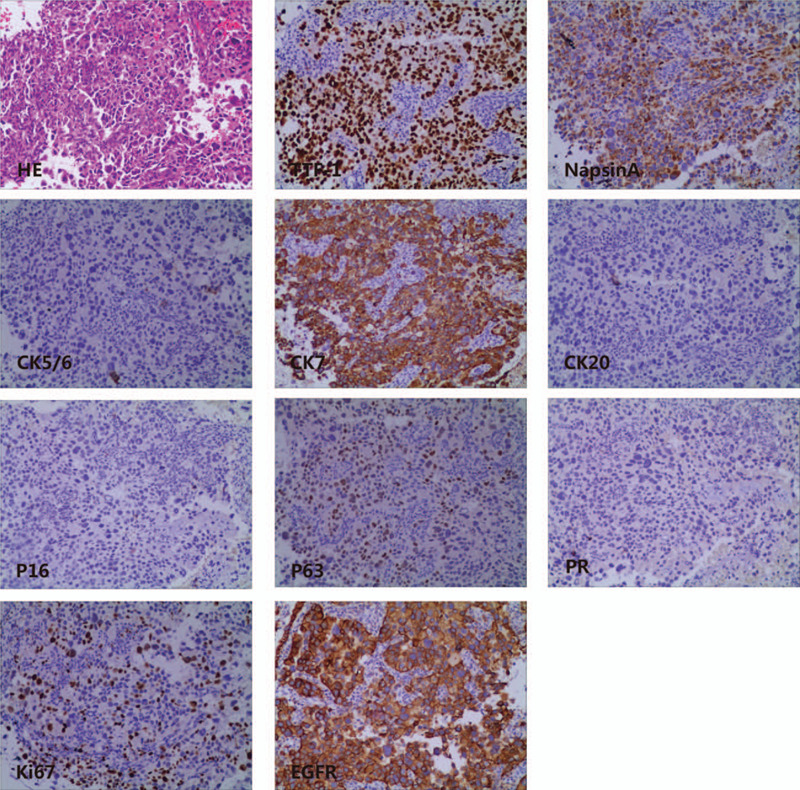
Endocervical curettage biopsy specimen hematoxylin-eosin staining shows poorly differentiated adenocarcinoma. Immunohistopathology indicates the presence of TTF-1, Napsin A, CK7 and Ki-67 (50%) and negative staining for CK5/6, CK20, P16, P63, and PR (magnification × 400).

**Figure 3:**
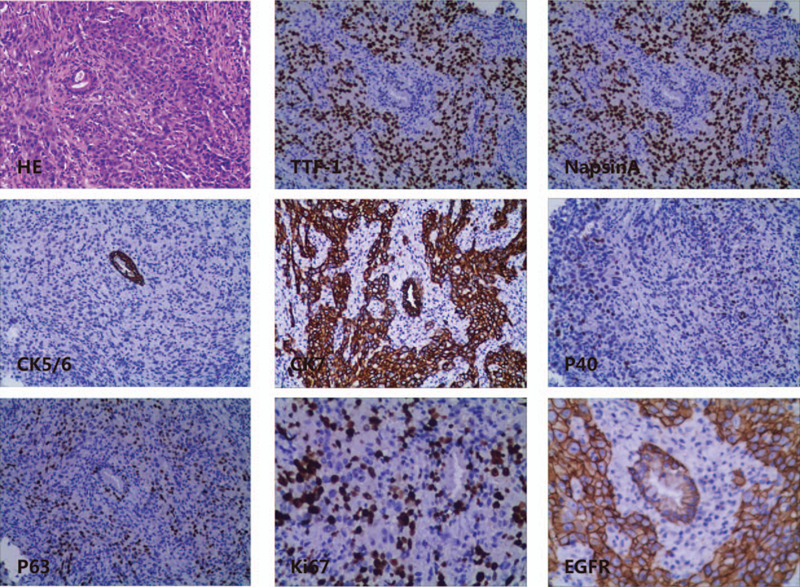
Lung biopsy specimen hematoxylin-eosin staining shows poorly differentiated adenocarcinoma. Immunohistopathology indicates the presence of TTF-1, Napsin A, CK7, EGFR and Ki-67 (40%) and negative staining for CK5/6, P40, and P63 (magnification × 400).

**Figure 4:**
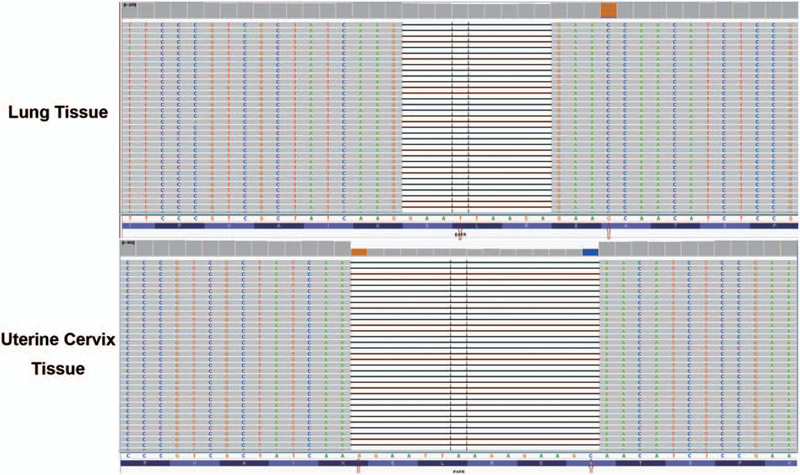
Next-generation sequencing (NGS) results of the primary lung tissue and metastatic uterine cervix tissue.

Magnetic resonance imaging (MRI) suggested cervical carcinoma (Fig. 1C and D). Metastases in the lumbar vertebra, sacrum, ilium, acetabulum, and femoral head as well as multiple bilateral iliac lymph nodes were observed. Gynecological colposcopy revealed uterine thickening in the cervix only at 12 o’clock, and no broccoli-like mass lesions were found in the cervix. Subsequently, intracervical curettage was performed for pathological examination, showing poorly differentiated cancer. Subsequent immunohistochemistry (IHC) analysis showed positive expression of thyroid transcription factor-1 (TTF-1), Napsin A, CK7 and Ki-67 (50%). There was no expression of CK5/6, CK20, P16, P63 or PR (Fig. 2). The high expression of TTF-1 and Napsin A suggested metastatic adenocarcinoma of the lung. These 2 markers are the most commonly used and represent the most valuable specific immunomarker combination for the diagnosis of lung adenocarcinoma to distinguish between primary and metastatic disease.^[1,2]^ Subsequent computed tomography (CT) of the chest and abdomen showed that the lesion was in the upper left lobe near the hilum of the lung (Fig. 1A and B). Additionally, there was accompanying left hilar lymph node and mediastinum enlargement, left adrenal thickening, and multi-rib bone and thoracolumbar metastasis. Brain MRI suggested right cerebellar hemisphere, left frontal lobe and meningeal metastases. Subsequent bronchoscopy revealed lung adenocarcinoma (Fig. 3). IHC staining showed high expression of TTF-1, Napsin A, CK7, EGFR, and Ki-67 (40%) and negative results for CK5/6, P40 and P63 (Fig. 3). The results of IHC staining showed that the lung lesions had high expression of TTF-1 and Napsin A with a lack of expression of CK5/6 and P40. The above IHC staining results were consistent with the intracervical biopsy results of lung metastatic adenocarcinoma. Based on the IHC staining results and CT imaging findings, the eventual diagnosis of the patient was primary pulmonary adenocarcinoma metastatic to the uterine cervix, stage IVb, cT2bN2M1c. We advised the patient to submit samples of DNA for next-generation sequencing from both the lung and cervical tumor biopsies. The results of next-generation sequencing revealed consistent EGFR mutations (EGFR exon 19 deletion) between the primary lung biopsy and metastatic uterine cervical biopsy specimens (Fig. 4).

Osimertinib (80 mg orally, once daily) was started for the patient in July 2019. Lumbago and sacroiliac joint pain were significantly relieved, and the ability to walk normally was achieved after a week. Levels of the tumor markers CEA, CA125, NSE, and CYFRA21-1 decreased significantly after 1 month and were within normal levels after 2 months of treatment (Table 1). Follow-up CT and MRI reexamination demonstrated that the primary pulmonary lesion and cervical metastases were markedly reduced, which was considered partial response (Fig. 1E-M). Bilateral iliac lymph node, hilar lymphadenopathy, left adrenal gland, and brain metastases diminished to different degrees. Symptoms of vaginal bleeding disappeared after osimertinib treatment, which was considered partial response (PR). The patient did not experience drug-related adverse effects during osimertinib treatment. The patient continues to take osimertinib at present.

**Table 1:**
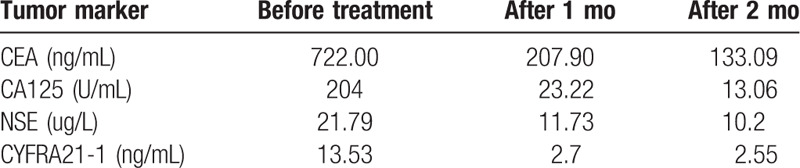
Tumor marker level of the patient after treatment.

## 3 Discussion

The brain, bone, liver and adrenal gland are the general organs likely of metastasis from NSCLC. However, the cervix is an unusual site for metastases from primary NSCLC. Metastases of the uterine cervix are often from female genital tumors, such as those of the ovaries and fallopian tubes. In general, genital tumors rarely metastasize to the cervix, and even if metastasis occurs, it is more common in gastric-related cancer, breast cancer and colorectal cancer. The case of metastasis from NSCLC to the cervix is very rare.^[3,4]^ Adenocarcinoma is a subtype of NSCLC that is the most frequent to metastasize.^[3,5]^ The reason why this rare phenomenon occurs is that the cervix is small in size, the blood flow is poor, and the cervix is filled with fibrous tissue, which is difficult to penetrate and grow. Because of these reasons, general genital metastases very rarely reach the cervix, and routine screening less commonly assesses the cervix; even if the cervix is assessed, cancer metastasis is very rare.^[3,6]^ Most of the symptoms are postmenopausal bleeding. For some young female patients, most physicians usually even consider that this is normal menstruation and neglect the possibility of metastasis. Appropriate pathological examination of both primary lesions and metastases is essential for definite diagnosis.^[7]^ It determines the selection of follow-up treatment strategies and prognosis.

Among previous treatment strategies, chemotherapy was the first treatment for advanced NSCLC. For example, in this report, 4 years after the patient's right upper lobe resection, primary lung adenocarcinoma was found to metastasize to the uterine cervix, and the patient selected first-line chemotherapy with carboplatin and paclitaxel.^[8]^ Currently, detecting the driver genes of advanced NSCLC is a necessary measure that is very important for the treatment and monitoring of tumors.^[9,10]^ For example, in this report, ALK rearrangement was detected in a patient with simultaneous cervical and mammary gland metastases from lung adenocarcinoma between the primary and metastatic cancers.^[11]^ Crizotinib was prescribed as a first-line treatment. In this case, EGFR gene mutations (Del19) were detected in both the primary lesion and metastatic site biopsy specimens. Osimertinib was chosen as the first-line treatment. The primary pulmonary lesions and metastatic sites of the uterine cervix, brain and other sites were significantly reduced.

In summary, lung adenocarcinoma metastatic to the uterine cervix is rare. Even so, cervical metastases from NSCLC should be considered. The main symptoms were vaginal bleeding. Cervical screening and immunohistochemical examination are particularly important for diagnosis and treatment. Gene detection in NSCLC and mutational analyses are necessary and may be useful for determining whether a tumor is primary or metastatic. Early and accurate diagnosis is of great significance for the treatment and prognosis of patients.

## Author contributions

**Conceptualization**: Yong Wang, Shubin Liu.

**Data curation:** Yong Wang, Lin Chen, Zhi Wang.

**Funding acquisition:** Yong Wang, Shubin Liu.

**Investigation:** Yong Wang, Shubin Liu.

**Visualization**: Shubin Liu.

**Writing – original draft**: Yong Wang, Lin Chen.

**Writing – review & editing**: Shubin Liu.
