# Lung adenocarcinoma with EGFR gene mutation metastatic to the uterine cervix: A case report: Erratum

**DOI:** 10.1097/MD.0000000000033976

**Published:** 2023-05-26

In the article “Lung adenocarcinoma with EGFR gene mutation metastatic to the uterine cervix: A case report,”^[1]^ there was a mistake in Figure 3 as published. Some of the images were interchanged. The corrected Figure 3 appears below. In addition, in the Figure 2 and 3 legends, the magnification was mistakenly listed as ×400. For both figures, the magnification is actually ×100.

**Figure:**
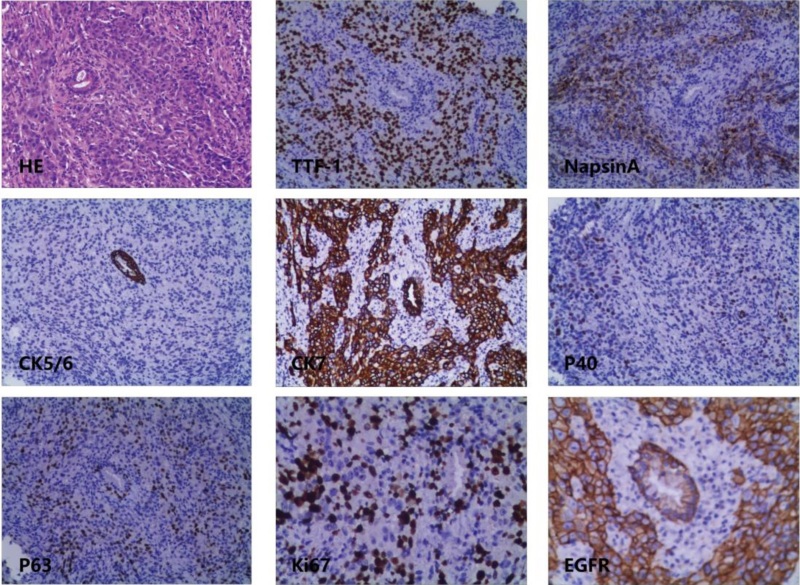


## References

[1] Wang Y Chen L Wang Z. Lung adenocarcinoma with EGFR gene mutation metastatic to the uterine cervix: a case report. Medicine (Baltimore). 2020;99:e22636. 33080701 10.1097/MD.0000000000022636 PMC7572012

